# Supplementary material for: Compliance with vessel speed restrictions to protect North Atlantic right whales
Source: PeerJ. 2014 Jun 3;2:e399. doi: 10.7717/peerj.399 (PMC4060020; doi:10.7717/peerj.399)
Supplement: Appendix S1 — See Silber & Bettridge (2012) for more complete descriptions. [file peerj-02-399-s001.docx]

**Supplemental Information, Appendix 1.** Outreach used to notify mariners of the December 2008 vessel speed restrictions. See Silber & Bettridge (2012) for more complete descriptions.

- *U.S. Coast Pilot.* **Issued by NOAA in nine regional volumes** and updated and published annually**, the** *Coast Pilot* **is a key navigational aid that contains precautionary notices, information on navigational hazards, voyage planning, and related data that are vital to safe navigation. Carrying the** U.S. *Coast* ***Pilot* and** knowledge of the information contained therein is mandatory for all vessels traveling in U.S. waters.
- *Maritime Broadcasts*. USCG Broadcast Notice to Mariners (marine radio broadcasts), Local Notice to Mariners (updated weekly and distributed electronically), NOAA maritime weather broadcasts, and notifications on NOAA Weather Buoy websites. These are key sources of information frequently consulted by prudent mariners sailing in U.S. waters; maritime accidents resulting from a lack of awareness of USCG broadcasts..
- *International Sailing Publications*. The National Geospatial Intelligence Agency’s *Notice to Mariners* and *Sailing Directions*, and to the United Kingdom’s *Admiralty Publications*. Updated and published annually, these international guidance documents are distributed broadly and are considered essential guides for mariners on international voyages.
- *Nautical Charts*. The management zones, their timing, and applicable restrictions were noted on printed nautical charts, a mainstay for navigational safety.
- *Mandatory Ship Reporting systems*. Under these systems, mariners are required, as a matter of port entry into U.S. ports, to report to a USCG shore-station when entering two key right whale aggregation areas (see Ward et al. 2005 for a description). Ships are then sent an automated message with whale locations and ways to reduce vessel strikes, including information about speed restrictions.
- *Informational Brochures*. Laminated two-page compliance guides distributed and posted on web sites. An estimated 3,000 guides were distributed by the USCG, port authorities, pilots, shipping industry liaisons, and others. http://www.nmfs.noaa.gov/pr/pdfs/shipstrike/compliance_guide.pdf
- *Captain and Crew Training Material*. A merchant mariner training curriculum designed primarily for maritime academies. Training through these academies is essential for those seeking captain’s licenses or upgrades to their license and the ship strike reduction curriculum is required in most U.S. academies. *Interactive CD-ROMs*. Captain and crew interactive training compact discs and “Right Whale Protection Program” notebooks were also developed. An estimated 2,500+ CDs and over 550 notebooks were distributed at no cost to the user.
- *Electronic Distribution Lists*. NMFS maintained electronic mail distribution lists for the shipping industry (containing several hundred recipients). Similar distributions were made via electronic mail lists (also containing owners and operators of several hundred vessels) maintained by industry associations, whereby their member companies were notified.
- *Agency and Company Information Dissemination*. Electronic mail notices were periodically and routinely distributed by the U.S. Maritime Administration (MARAD) to its entire fleet of 5,000+ domestically-flagged vessels. MARAD maintains or has jurisdiction over these commercially-operated material transport and cargo vessels that are pressed into service in time of war or national emergency as naval auxiliaries. A similar number of vessels were routinely notified about the requirements by Lloyds Registry.
- *Popular Press*. Press releases issued and numerous stories appeared in industry trade journals, local and national newspapers, and in radio spots.
